# Supplementary material for: Factors Associated with Dietary Diversity in Community-Dwelling Brazilian Older Adults
Source: Foods. 2024 Oct 29;13(21):3449. doi: 10.3390/foods13213449 (PMC11545754; doi:10.3390/foods13213449)
Supplement: Supplementary file 1 [file foods-13-03449-s001.zip › foods-3058390-supplementary.pdf]

Supplementary File 1. Survey questionnaire (in Brazilian Portuguese)

Questionário da pesquisa

Nome entrevistador: ____

Nome do idoso:____
Gênero: (   ) Masculino   (   ) Feminino   Data de Nascimento:__/__/___
Endereço:____

____

____

Telefone do domicílio: (   )______________________________
E-mail:____
Nome e Telefone de um familiar, amigo ou vizinho para contato: ________________________
Telefone (   )____

BLOCO A - IDENTIFICAÇÃO E CARACTERÍSTICAS SÓCIO DEMOGRÁFICAS

A1 - Estado Civil

1. Solteiro
2. Casado(a) ou vive com companheiro(a)
3. Divorciado(a), separado(a) ou desquitado(a)
4. Viúvo(a)
8. NS
9. NR

A2 - O(a) Sr.(a) foi à escola?

1. Sim
2. Não
3. Nunca frequentou, mas sabe ler e escrever
4. Nunca frequentou, não sabe ler e escrever   **(<u>PULAR para A4</u>)**
8. NS
9. NR

A3 – Quantos anos de estudo você teve? ________________ (em números de anos)
{ANOTE A SÉRIE DO ÚLTIMO GRAU APROVADO}

- Primeiro grau (ou primário + ginásio). Considerar de 1 a 8 anos.
- Segundo grau (antigo clássico e científico). Considerar de 9 a 11 anos.
- Técnico de nível médio (ex.: técnico em contabilidade, laboratório). Considerar de 9 a 11 anos.
- Magistério – segundo grau (antigo normal). Considerar de 9 a 11 anos.
- Graduação (nível superior). Considerar 15 anos ou somar o tempo que cursou.
- Supletivo/Madureza. Considerar de 9 a 11 anos.
- NS
- NR

A4 - Qual cor de pele o descreve melhor?

1. Branco
2. Pardo /mulato / moreno (combinação de branco e preto)
3. Preto
4. Indígena
5. Amarelo
6. Outra (especificar) ___________________
8. NS
9. NR

A5 - O senhor(a) é a pessoa responsável (chefe de família)?

1. Sim
2. Não, esposa(o)
3. Não, filho(a)
4. Não, genro/nora
5. Não, outro
8. NS
9. NR

A6 - Quantos filhos o(a) Sr.(a) tem? _____ 88.NS 99.NR

A7 - Quantos filhos vivos o(a) Sr.(a) tem? _____ 88.NS 99.NR

A8 – Além de você, quantas pessoas moram no seu domicílio?________ 88.NS 99.NR

A9 - O(a) senhor(a) pode detalhar quem são estas pessoas que moram com você?
(OBS: marque o parentesco de acordo com a legenda abaixo)

1. Mora sozinho
2. Cônjuge
3. Pai/Mãe
4. Filho (os)
5. Irmão (s)
6. Neto (s)
7. Tio/Tia
8. Genro/Nora
9. Cunhado (a)
10. Amigo (s)
11. Outro Familiar
12. Outro não familiar

NOME	PARENTESCO (código)	IDADE

A10 - O Sr.(a) exerce algum trabalho remunerado atualmente?
1. Sim 2. Não **(<u>PULAR para</u> A12)** 8.NS 9.NR

A11 - Qual a sua ocupação atual? ____
8.NS 9.NR

A12 - Qual a sua ocupação anterior? ____
8.NS 9.NR

A13-Qual a sua renda mensal individual? R$________________(em valor bruto)
8.NS 9.NR

A14 - Qual a renda total das pessoas que moram com o(a) senhor(a)m incluindo-o(a)? R$______________________(em valor bruto) 8.NS 9.NR

**A15 - Quais as fontes provenientes dessa renda**:	**(1) Idoso**	**(2) Familiar**	**(9) NR**
Aposentadoria			
Pensão			
BPC (benefício de prestação continuada)			
Aluguel de uma propriedade			
Salário			
Auxílio do governo (bolsa família..)			

BLOCO B – COGNIÇÃO, DEPRESSÃO E APOIO FAMILIAR
APLICAR SOMENTE AO IDOSO

B1 - Instrução: "Eu vou examinar sua memória. Vou dizer três nomes para você repetir e gravar na memória para lembrar depois. Repita os nomes somente depois que eu tiver falado todos os três." (Diga com clareza os três nomes, aproximadamente 1 nome por 1.5 segundo. Dê 1 ponto para cada nome repetido corretamente após a primeira apresentação oral dos mesmos. Os nomes podem ser reapresentados até três vezes.)

CAMISA MARROM HONESTIDADE

PONTUAÇÃO (total de nomes repetidos – 0 se o idoso não repetir nenhum) ________

ORIENTAÇÃO NO TEMPO – Marque a pontuação correspondente à alternativa de acordo com a resposta do (a) idoso (a)

B2 - "Em que ano nós estamos?" RESPOSTA__________PONTUAÇÃO _________

Ano correto - 4 pontos	Erra por 2 a 5 anos – 1 ponto
Erra por 1 ano – 2 pontos	Erra por 6 ou mais anos – 0 ponto

B3 - "**Qual é o mês e o dia-do-mês em que estamos?" RESPOSTA_PONTUAÇÃO ______________**

Resposta correta - 5 pontos	Erra por 6 a 29 dias- 2 pontos
Erra por 1 a 2 dias - 4 pontos	Erra por 30 a 59 dias - 1 ponto
Erra por 3 a 5 dias - 3 pontos	Erra por 60 ou mais dias - 0 ponto

B4 - "Que dia da semana é hoje?" RESPOSTA_____________PONTUAÇÃO _______

Resposta correta - 1 ponto	Resposta errada- 0 ponto

B5 - "Que horário do dia é agora?" RESPOSTA________PONTUAÇÃO ________

Resposta exata ou com erro de até 60 minutos - 1 ponto	Resposta errada- 0 ponto

B6 - FLUÊNCIA VERBAL: "Agora eu quero que você diga os nomes de todos os animais de quatro pernas que você conhece. Você vai ter 30 segundos para dizer o máximo de nomes que você lembrar. Pode começar." (Dê 1 ponto para cada resposta correta, até um máximo de 10 pontos). PONTUAÇÃO (0 – 10)

B7 - EVOCAÇÃO: “Você se lembra daqueles 3 nomes que eu pedi para você guardar na memória?”
RESPOSTA :__________PONTUAÇÃO : ______________________

**A (CAMISA)** Evocação espontânea (sem ajuda)	3 pontos
Se após: “Um dos nomes era de uma coisa que usamos no corpo”	2 pontos
Se após: “Um dos nomes era sapatos, camisa ou meias?”	1 ponto
Se mesmo com estas dicas continua incapaz de lembrar	0 ponto

**B (MARROM)** Evocação espontânea	3 pontos
Se após: “Uma das palavras era o nome de uma cor”	2 pontos
Se após: “Um dos nomes era azul, preto ou marrom?”	1 ponto
Se mesmo com estas dicas continua incapaz de lembrar	0 ponto

**C (HONESTIDADE)** Evocação espontânea	3 pontos
Se após: “Um dos nomes se referia a uma boa qualidade pessoal”	2 pontos
Se após: “Um dos nomes era honestidade, caridade ou modéstia?”	1 ponto
Se mesmo com estas dicas continua incapaz de lembrar	0 ponto

Agora pense nas últimas duas semanas e diga como se sentiu na maior parte do tempo nesse período...

	**Sim**	**Não**	**NS**	**NR**
**B8 - O(a) Sr.(a) está basicamente satisfeito com a sua vida?**	1	2	8	9
**B9 - Tem diminuído ou abandonado muitos dos seus interesses ou atividades anteriores?**	1	2	8	9
**B10 - Sente que sua vida está vazia?**	1	2	8	9
**B11 - Tem estado aborrecido frequentemente?**	1	2	8	9
**B12 - Tem estado de bom humor a maior parte do tempo?**	1	2	8	9
**B13 - Tem estado preocupado ou tem medo de que alguma coisa ruim vá lhe acontecer?**	1	2	8	9
**B14 - Sente-se feliz a maior parte do tempo?**	1	2	8	9
**B15 - Com frequência se sente desamparado ou desvalido?**	1	2	8	9
**B16 - Tem preferido ficar em casa em vez de sair e fazer coisas?**	1	2	8	9
**B17 - Tem sentido que tem mais problemas com a memória do que outras pessoas de sua idade?**	1	2	8	9
**B18 - O(a) sr(a) acredita que é maravilhoso estar vivo?**	1	2	8	9
**B19 - Sente-se inútil ou desvalorizado em sua situação atual?**	1	2	8	9
**B20 - Sente-se cheio de energia?**	1	2	8	9
**B21 - Se sente sem esperança diante da sua situação atual?**	1	2	8	9
**B22 - O(a) sr(a) acredita que as outras pessoas estão em situação melhor?**	1	2	8	9

Aproximadamente, quantos amigos ou familiares próximos o senhor(a) têm? (Pessoas com as quais você fica à vontade e pode falar de tudo o que quiser. Pessoas com quem você pode contar quando precisa de ajuda).

B23 - Escreva o número de amigos e familiares próximos: ___________

**B24 - Pense nas pessoas com as quais o senhor(a) fica à vontade, pode falar de tudo o que quiser e pode contar quando precisa de ajuda.**<br>No geral, essas pessoas são:	1. Familiares que moram com o senhor(a)<br>2. Familiares que não moram com o senhor(a)<br>3. Amigos e/ou vizinhos<br>8. NS<br>9. NR
**B25 - A maior parte dos seus amigos e familiares próximos é homem ou mulher?**	1. Mesmo número de homens e de mulheres<br>2. Maioria mulheres<br>3. Maioria homens<br>8. NS<br>9. NR
**B26 - Maior parte dos seus amigos e familiares próximos é criança/adolescente, adulto ou idoso?**	1. Maioria criança/adolescente<br>2. Maioria adulto<br>3. Maioria idoso<br>8. NS<br>9. NR
**B27 - O senhor(a) mora com alguma criança? (não somente netos, mas outras crianças também)**	1. Sim<br>2. Não<br>8. NS<br>9. NR
**B28 - O senhor mora apenas com outro(s) idoso(s)?**	1. Sim<br>2. Não<br>8. NS<br>9. NR
**B29 - No geral, com que frequência o senhor(a) tem contato com a maioria dos seus amigos e familiares próximos?**	1. Nunca 5. Anualmente<br>2. Diariamente 8. NS<br>3. Semanalmente 9. NR<br>4. Mensalmente
**B30 - No geral, como o senhor(a) se sente em relação ao contato com a maioria dos seus amigos e familiares próximos?**	1. Muito satisfeito 4. Nada satisfeito<br>2. Satisfeito 8.NS<br>3. Pouco satisfeito 9. NR

Caso o entrevistado responda **SIM** a pergunta colocar com que frequência →	**Raramente**	**Às vezes**	**Quase sempre**	**Sempre**
**B31.Você tem alguém que o ajude se estiver doente, de cama?** (1) Sim (2) Não	1	2	3	4
**B32. Você tem alguém para lhe ouvir quando você precisa falar?** (1) Sim (2) Não	1	2	3	4
**B33. Você tem alguém para lhe dar bons conselhos em uma situação de crise?** (1) Sim (2) Não	1	2	3	4
**B34 - Você tem alguém para levá-lo ao médico?** (1) Sim (2) Não	1	2	3	4
**B35 -Você tem alguém que demonstre amor e afeto por você?** (1) Sim (2) Não	1	2	3	4
**B36 - Você tem alguém para se divertir junto?** (1) Sim (2) Não	1	2	3	4
**B37 - Você tem alguém para lhe dar uma informação que o ajude a compreender deter minada situação?** (1) Sim (2) Não	1	2	3	4

Continuando...	Raramente	Às vezes	Quase sempre	Sempre
**B38 - Você tem alguém em quem confiar para falar de você ou sobre seus problemas?** (1) Sim (2) Não	1	2	3	4
**B39 - Você tem alguém que lhe dê um abraço?** (1) Sim (2) Não	1	2	3	4
**B40 - Você tem alguém com quem relaxar?** (1) Sim (2) Não	1	2	3	4
**B41 - Você tem alguém para preparar suas refeições se você não puder prepará-las?** (1) Sim (2) Não	1	2	3	4
**B42 - Você tem alguém de quem você realmente quer conselhos?** (1) Sim (2) Não	1	2	3	4
**B43 - Você tem alguém com quem distrair a cabeça?** (1) Sim (2) Não	1	2	3	4
**B44 - Você tem alguém para ajudá-lo nas tarefas diárias se você ficar doente?** (1) Sim (2) Não	1	2	3	4
**B45 - Você tem alguém para compartilhar suas preocupações e medos mais íntimos?** (1) Sim (2) Não	1	2	3	4
**B46 - Você tem alguém para dar sugestões de como lidar com um problema pessoal?** (1) Sim (2) Não	1	2	3	4
**B47 - Você tem alguém com quem fazer coisas agradáveis?** (1) Sim (2) Não	1	2	3	4
**B48 - Você tem alguém que compreenda seus problemas?** (1) Sim (2) Não	1	2	3	4
**B49 - Você tem alguém que você ame e que faça você se sentir querido?** (1) Sim (2) Não	1	2	3	4

BLOCO C - ESTADO DE SAÚDE

C1 - O(a) Sr(a) fuma atualmente ?
1.Sim 2.Não (**PULAR para C4**) 8.NS 9.NR

C2 - Há quanto tempo é fumante (anos)?__________8.NS 9.NR

C3 - Quantos cigarros fuma por dia ?____________8.NS 9.NR

C4 - Já fumou e largou ? 1.Sim 2.Não (**PULAR para C6**) 8.NS 9.NR

C5 - Há quanto tempo parou de fumar?______meses ______anos 8.NS 9.NR

C6 - Com que frequência o(a) Sr(a) consome bebidas alcóolicas ?
1. Nunca **(PULAR PARA C8)**
2. Raramente
3. Uma vez por mês ou menos
4. De 2 a 4 vezes por mês
5. De 2 a 3 vezes por semana
6. De 4 a 7 vezes por semana
8. NS
9. NR

C7 - Quantas doses de álcool o/a senhor/a consome em um dia normal?
1. Uma dose
2. Duas ou três doses
3. Quatro ou cinco doses
4. Seis ou sete doses
5. Oito ou mais
8. NS
9.NR

C8 - O (a) Sr. (a) já bebeu e parou? (SOMENTE PARA QUEM RESPONDEU 1 NA C6)
1.Sim 2.Não 8.NS 9.NR

C9 - Há quanto tempo o(a) sr(a) parou de beber ?________ 8.NS 9.NR

C10 - Por que parou de beber (deixar o idoso responder e marcar a melhor alternativa)
1. Doença / problema de saúde exigiu restrição no consumo (Médico ou outro profissional orientou)
2. Leu ou assistiu a alguma reportagem / programa que falava dos males causados
3. Achou melhor para a saúde
4. Parentes / amigos recomendaram
5. Outros____

8.NS
9.NR

Algum médico ou outro profissional de saúde já disse que o (a) Sr(a) tem alguma das seguintes doenças ou problemas de saúde? Se sim, esta doença limita ou não limita as suas atividades do dia-a-dia? Você toma remédio para controlar este problema?	**A**.Diagnóstico				**B**.Limitação				**C**.Remédio			
	**Sim**	**Não**	**NS**	**NR**	**Sim**	**Não**	**NS**	**NR**	**Sim**	**Não**	**NS**	**NR**
**C11 - Hipertensão**	1	2	8	9	1	2	8	9	1	2	8	9
**C12 – Diabetes**	1	2	8	9	1	2	8	9	1	2	8	9
**C13 - Doença cardiovascular**	1	2	8	9	1	2	8	9	1	2	8	9
**C14 - Tumor/Câncer**	1	2	8	9	1	2	8	9	1	2	8	9
**C15 - AVC ou derrame**	1	2	8	9	1	2	8	9	1	2	8	9
**C16 - Doença Crônica Pulmonar (asma, enfisema, etc)**	1	2	8	9	1	2	8	9	1	2	8	9
**C17 - Reumatismo/Artrite/ Artrose**	1	2	8	9	1	2	8	9	1	2	8	9
**C18 - Osteoporose**	1	2	8	9	1	2	8	9	1	2	8	9
**C19 - Dor de cabeça frequente/ Enxaqueca**	1	2	8	9	1	2	8	9	1	2	8	9
**C20 - Dor nas costas/Problema na coluna**	1	2	8	9	1	2	8	9	1	2	8	9
**C21 - Alergia: _________**	1	2	8	9	1	2	8	9	1	2	8	9
**C22 - Problema Emocional (depressão/ ansiedade/tristeza)**	1	2	8	9	1	2	8	9	1	2	8	9
**C23 - Tontura/Vertigem**	1	2	8	9	1	2	8	9	1	2	8	9
**C24 – Doenças renais/Infecção Urinária**	1	2	8	9	1	2	8	9	1	2	8	9

	A.Diagnóstico				B.Limitação				C.Remédio			
	**Sim**	**Não**	**NS**	**NR**	**Sim**	**Não**	**NS**	**NR**	**Sim**	**Não**	**NS**	**NR**
**C25 - Deficiência Auditiva**<br>tipo 1.deficiência 2.surdez 3.perda	1	2	8	9	1	2	8	9	1	2	8	9
**C26 - Deficiência Visual**<br>tipo 1.deficiência 2.cegueira um olho<br>3. cegueira dois olhos	1	2	8	9	1	2	8	9	1	2	8	9
**C27 - Outros :**	1	2	8	9	1	2	8	9	1	2	8	9

Nos últimos 12 meses o(a) Sr. (a) teve algum destes problemas?	**Sim**	**Não**	**NS**	**NR**
**C28 -** Incontinência urinária (ou perda involuntária da urina)	1	2	8	9
**C29 -** Incontinência fecal (ou perda involuntária das fezes)	1	2	8	9
**C30-** Dificuldade de memória, de lembrar-se de fatos recentes	1	2	8	9
**C31-** Lesões de pele, feridas ou escaras	1	2	8	9
**C32 -** Alguma dor constante ou que vai e vem	1	2	8	9

C33 – Quantos medicamentos o (a) Sr. (a) utiliza atualmente de maneira regular? ____________________

C34 - Como é o seu hábito intestinal ?

1. Normal
2. Constipado (preso)
3. Diarréico
4. Variado
8. NS
9. NR

C35 - Qual a frequência de evacuação?_____vezes ao 1. Dia 2. Semana 8.NS 9. NR

C36 - De um modo geral, como o/a senhor/a avalia a sua saúde no momento atual?

1<br>Muito Ruim	2<br>Ruim	3<br>Regular	4<br>Boa	5<br>Muito Boa	8<br>NS	9<br>NR

C37 - Como o/a senhor/a avalia sua saúde em comparação com a saúde de outras pessoas da sua idade?

1<br>Muito Pior	2<br>Pior	3<br>Igual	4<br>Melhor	5<br>Muito Melhor	8<br>NS	9<br>NR

C38 – Como o/a senhor/a avalia a sua saúde hoje em comparação com a de 1 ano atrás?

1<br>Muito Pior	2<br>Pior	3<br>Igual	4<br>Melhor	5<br>Muito Melhor	8<br>NS	9<br>NR

ATIVIDADE FÍSICA

**Agora, vou dizer o nome de algumas atividades físicas que as pessoas realizam por prazer, para se exercitar, para se divertir, porque fazem bem para a saúde ou porque precisam. Gostaria que me dissesse se costuma realizar essas atividades, em quantos dias na semana e quanto tempo por dia.**	**A.**Na última semana:<br>1. Sim<br>2. Não<br>8. NS<br>9. NR	**B**. Qtos dias/ semana ?	**C**. Tempo/dia (min)	**D.** Intensidade<br>1. Leve<br>2. Moderada<br>3. Vigorosa
**C44 -** Faz caminhadas como forma de exercício?				
**C45 -** Pratica corrida leve ou caminhada vigorosa?				
**C46 -** Faz ginástica, yoga, tai-chi-chuan ou outra atividade desse tipo?				
**C48** - Faz musculação?				
**C49 –** Faz hidroginástica ou natação?				
**C50-** Pratica algum outro tipo de exercício físico ou esporte que eu não mencionei?	**QUAL?** ______________________________			

EXPOSIÇÃO SOLAR

C51 - Você tem o hábito de se expor ao sol para alguma(s) das atividades abaixo?
1. Atividades de lazer (jardinagem, praças, parques).
2. Para realizar atividade física (caminhada, corrida, etc...).
3. Durante percursos do dia-a-dia (ir até o ponto de ônibus, ao mercado, feiras, etc...).
4. Com a finalidade de ter mais saúde.
8. NS
9. NR

C52 - Tem o hábito de usar protetor solar nesses momentos?
1. Sim   2. Não   8. NS   9. NR

C53 - Qual a frequência da exposição solar?
1. < 3 dias por semana   2. >= 3 dias por semana   8. NS   9. NR

C54 - E média, quanto tempo dura a exposição diária?
1. < 15 minutos   2. >= 15 minutos   8. NS   9. NR

C55 - Qual horário do dia costuma se expor ao sol?
1. Antes das 9h   2. Das 9-15h   3. Após 15h   8. NS   9. NR

C56 - Quais as partes do seu corpo costumam ficar expostas ao sol?
1. Rosto (certificar que sem uso de chapéu/boné)
2. Mãos
3. Braços
4. Pernas
8. NS
9. NR

C57- Escala de Fitzpatrick (1976) (ver escala impressa)
1. Tipo 1
2. Tipo 2
3. Tipo 3
4. Tipo 4
5. Tipo 5
6. Tipo 6

BLOCO D - SARCOPENIA, FRAGILIDADE E QUEDAS

D1 - Quanta dificuldade tem para levantar ou carregar 4,5 kg (um saco de arroz)?
0. Nenhuma 8. NS
1. Alguma 9. NR
2. Muita ou não consegue

D2 - Quanta dificuldade tem para andar dentro de um cômodo?
0. Nenhuma 8. NS
1. Alguma 9. NR
2. Muita, usa equipamento ou não consegue

D3 - Quanta dificuldade tem para levantar de uma cadeira ou cama?
0. Nenhuma 8. NS
1. Alguma 9. NR
2. Muita ou não consegue

D4 - Quanta dificuldade tem para subir 10 degraus de escada (1 lance)?
0. Nenhuma 8. NS
1. Alguma 9. NR
2. Muita ou não consegue

D5 - Quantas vezes caiu no último ano?
0. Nenhuma (**<u>PULAR PARA D9</u>**)
1. Uma
2. Duas
3. Três
4. Quatro ou mais
8. NS
9. NR

D6- Quando foi a sua última queda?
1. Há menos de 15 dias
2. 15 a 30 dias
3. 30 a 90 dias
4. Há mais de 90 dias
8. NS
9. NR

D7- Por causa dessa (as) queda (as) o(a) Sr(a) teve alguma fratura?
1. Sim 2. Não (**<u>PULAR PARA D9</u>**) 8.NS 9. NR

D8- Em que local?
1. Mão
2. Braço
3. Perna
4. Quadril
5. Outro (qual) ____________

D9 - Nos últimos 12 meses, o(a) sr.(a) perdeu peso sem fazer nenhuma dieta? Sim, quantos quilos?
1. Entre 1 kg e 3 kg 8. NS
2. Mais de 3 kg 9.NR
3. Não perdeu peso

D10 - Nos últimos 12 meses (último ano), o(a) sr.(a) sente mais enfraquecido, acha que sua força diminuiu?
1.Sim 2. Não 8. NS 9. NR

D11 - O(A) sr.(a) acha que hoje está caminhando mais devagar do que caminhava há 12 meses (há um ano)?
1.Sim 2. Não 8. NS 9. NR

D12 - O(A) sr.(a) acha que faz menos atividades físicas do que fazia há 12 meses (há um ano)?
1.Sim 2. Não 8. NS 9. NR

D13 - Com que frequência, na última semana, o(a) sr.(a) sentiu que não conseguiria levar adiante suas coisas (iniciava alguma coisa mas não conseguia terminar):
1. Nunca ou raramente (menos de 1 dia)
2. Poucas vezes (1 - 2 dias)
3. Algumas vezes (3 - 4 dias)
4. A maior parte do tempo
8. NS
9. NR

D14 - Com que frequência, na última semana, a realização de suas atividades rotineiras exigiram do(a) sr.(a) um grande esforço para serem realizadas:
1. Nunca ou raramente (menos de 1 dia)
2. Poucas vezes (1 - 2 dias)
3. Algumas vezes (3 - 4 dias)
4. A maior parte do tempo
8. NS
9. NR

BLOCO E - NUTRIÇÃO

QNSA – Questionário Nutricional Simplificado de Apetite

**E1 - MEU APETITE ESTÁ:**		
1. Muito ruim<br>2. Ruim<br>3. Moderado	4. Bom<br>5. Muito bom	8. NS<br>9. NR
**E2 - QUANDO EU COMO:**		
1. Me sinto satisfeito após comer poucas garfadas/colheradas<br>2. Me sinto satisfeito após comer aproximadamente 1/3 da refeição<br>3. Me sinto satisfeito após comer mais da metade da refeição	4. Me sinto satisfeito após comer a maior parte da refeição<br>5. Dificilmente me sinto satisfeito	8. NS<br>9. NR
**E3 - O SABOR DA COMIDA É:**		
1. Muito ruim<br>2. Ruim<br>3. Mediano	4. Bom<br>5. Muito bom	8. NS<br>9. NR
**E4 - NORMALMENTE EU COMO:**		
1. Menos de uma refeição por dia<br>2. Uma refeição por dia<br>3. Duas refeições por dia	4. Três refeições por dia<br>5. Mais de três refeições por dia	8. NS<br>9. NR

SAÚDE BUCAL

O (a) Senhor (a) usa dentadura:	Sim	Não	NS	NR
**E5 - Na arcada superior?**	1	2	8	9
**E6 - Na arcada inferior?**	1	2	8	9
**E7 - A dentadura machuca ou cai?**	1	2	8	9
**E8 - Costuma alimentar-se com dentadura?**	1	2	8	9
**E9 - Tem sentido sua boca seca nas últimas semanas?**	1	2	8	9

E10 - Como o (a) senhor (a) avalia sua saúde bucal?

1<br>Muito Ruim	2<br>Ruim	3<br>Regular	4<br>Boa	5<br>Muito Boa	8<br>NS	9<br>NR

O (a) senhor (a) consome :
**E11 -** (MAN) **Pelo menos uma porção** diária **de leite ou derivados, tais como queijo e iogurte ?**<br>1. Sim 2. Não
**E12 -** (MAN) **Algum tipo de carne, peixe e aves** todos os dias **?**<br>1. Sim 2. Não
**E13 -** (MAN) **Duas ou mais porções** diárias **de fruta, verduras e legumes?**<br>1. Sim 2. Não
**E14 -** (MAN) **Duas ou mais porções** semanais **de leguminosas (feijão, ervilha ou soja) ou ovos?**<br>1. Sim 2. Não
**E15 -** (MAN) Nos últimos 3 meses**, o/a senhor/a percebeu que passou a comer menos, devido a perda de apetite, problemas digestivos ou dificuldade para mastigar ou deglutir/engolir ?**<br>0. Diminuição grave da ingesta<br>1. Diminuição moderada da ingesta<br>2. Sem diminuição da ingesta
**E16 -** (MAN) **Perda de peso nos últimos 3 meses:**<br>0. Superior a três quilos<br>1. Não sabe informar<br>2. Entre um e três quilos<br>3. Sem perda de peso
**E17 -** (MAN) **O (a) senhor (a) passou por algum estresse psicológico ou doença aguda** nos últimos 3 meses**?**<br>0. Sim<br>2. Não
**E18 -** (MAN) **O senhor acha que está desnutrido?**<br>0. Acredita estar desnutrido<br>1. Não sabe dizer<br>2. Acredita não ter um problema nutricional
**E19 -** (MAN) **Modo de se alimentar**<br>0. Não é capaz de se alimentar sozinho<br>1. Alimenta-se sozinho, porém com dificuldade<br>2. Alimenta-se sozinho sem dificuldade

	Número	NS	NR
**E20 -** (MAN) **Quantas refeições o (a) senhor (a) faz por dia (café da manhã, almoço, jantar)?**		88	99
**E21 -** (FIBRA) **Quantos lanches entre as refeições faz por dia?**		88	99
**E22 - Quantos copos de líquidos (água, suco, chá, leite) consome por dia?**		88	99
